# Non-Uniformity of the Indoor Radon Concentration under a Convective Mixing Mechanism

**DOI:** 10.3390/ijerph15122826

**Published:** 2018-12-11

**Authors:** Sergey Spotar, Nurlan Ibrayev, Aigerim Uyzbayeva, Jamil Atabayev

**Affiliations:** 1 School of Engineering, Nazarbayev University (NU), 53, Kabanbay Batyr Avenue, Astana 010000, Kazakhstan; sergey.spotar@nu.edu.kz (S.S.); jamil.atabayev@nu.edu.kz (J.A.); 2 National Laboratory Astana, Private Institution (NLA, PI), Nazarbayev University, 53, Kabanbay Batyr Avenue, Astana 010000, Kazakhstan; nurlan.ibrayev@nu.edu.kz

**Keywords:** radon, concentration, CFD, convection, diffusion

## Abstract

This paper focuses on the results of computational fluid dynamics (CFD) modeling of radon concentration distribution in living areas within residences. The COMSOL Multiphysics^®^ 5.3 software package has been employed for solving coupled momentum and species transport problems together with pseudo-reaction term modeling of the radon radioactive decay process. The reliability and verification of the simulation model was tested by comparing with available experimental data. The obtained results show the existence of stagnant zones where the concentration of radon is substantially higher than the average values. The impact of factors such as wind velocity, air tightness, and incoming radon flux were taken into consideration.

## 1. Introduction

Currently it is considered that exposure to radon gas is the one of the primary causes of lung cancer, and the most important one for non-smokers. In particular, there is a serious hazard resulting from radon gases that are concentrated in the foundations of buildings and poorly ventilated areas [1]. Being a product of uranium decay, radon is released either from building materials [2,3] or from underground [1,4]. This paper focuses on the details of the mechanism of convection mixing in living areas that might cause pronounced non-uniformity of radon concentrations in buildings.

## 2. Literature Review

The majority of researchers were concentrated on temporal behavior of the radon concentration in the houses [1,3,4] which assumes the complete mixing of radon entering into the building, i.e., in the continuity equation that describes the conservation of radon species [5]:

(1) $$\frac{dc\;\left(t\right)}{dt}+\lambda \left(t\right)c\left(t\right)=S\left(t\right)$$

where *c*, *λ*, and *S* are the radon concentration, air change rate, and radon entry rate, respectively. Consequently, it is assumed that the space can be treated as a single zone and that the variables are not in function of position. In other words, the models based on this approach operate with the average level of radon in the room.

The model is convenient, robust, and allows estimating the impact of many governing factors including transient ones (seasonal radon source strength changes, diurnal variations, meteorological data, etc.). However, the experimental observations and results of recent computational fluid dynamics (CFD) research papers [2,6,7] demonstrate that radon concentration in living areas might markedly deviate from average values. Herewith, it is also worth including in the analysis of the entire process those studies focusing on the modeling of the impact of the external environment (i.e., atmospheric values such as air wind velocity, permeability as a characteristic of soil) on radon entry into houses [8,9]. The variety and interplay of different factors affecting the level of radon concentration in living areas motivate researchers to further scrutinize the details of the phenomena.

## 3. Problem Definition/Formulation

The concentration of radon indoors mainly depends on its diffusion and the mixing capacity of convection, which being combined together with a variety of boundary conditions, might result in a substantially non-uniform distribution of the radon species in buildings. Computational fluid dynamics (CFD) can be efficiently applied as a valuable tool for research in this area [2,6,7].

**Figure 1:**
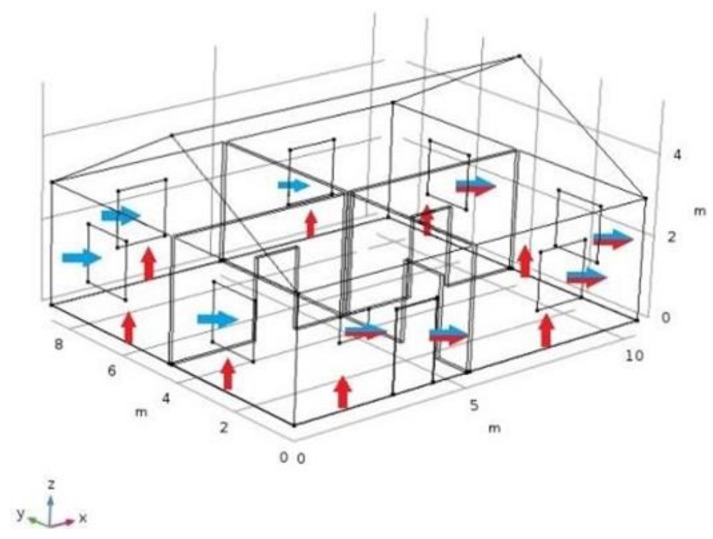
Geometry of the model. Schematic of the air streams and radon entry. (Legend: blue arrows—atmospheric air infiltrating the house due to the wind; red arrows—radon entry through the cracks; red and blue arrows—exiting streams on the leeward side).

The objective of this paper is to present the application of CFD techniques to study the effect of such factors as air exchange rates and airtightness of enclosing structures on the concentration of radon indoors. Herewith, we created a model of the house of the same shape and size as in [8,9] in order to employ the outcomes of a detailed study of the impact of the wind-induced entry of radon into the house with a specified geometry. Riley et al. [8,9] in their study considered this house as a ‘solid body’ object when they targeted the pressures and flow streamlines in the surrounding soil; we added the windows, simplified interior structures, and suggested the existence of cracks between the floor and walls. The schematic of air streams which determine the ventilation of the house and fluxes of radon entry is shown in Figure 1.

We included a wind velocity of 8.3 m∙s^−1^ in this case, which induced about 11 Pa of the soil–house pressure drop [8,9].

Adoption of the typical values of rural house airtightness [4] yields the air exchange rate input (λ) values for our model: 0.17 h^−1^, 0.34 h^−1^, and 0.51 h^−1^. As to the volumetric rate of the radon carrying air, application of the quadratic model for the crack flow [10] resulted in 7.54 × 10^−3^ m^3^ s^−1^ radon-carrying airflow. As to radon concentration in this inflow, it was taken to be 500 Bq∙m^−3^, which corresponds to territories characterized by the increased values of underground radon flux [11].

## 4. Method of Analysis

The COMSOL Multiphysics^®^ v.5.3 software package (COMSOL, Inc., Stockholm, Sweden) [12] has been used for the numerical implementation of the model with the following setting of the options and parameters.

Physics: ‘Turbulent Flow’ or ‘Laminar Flow’ and ‘Transport of Diluted Species’. Mesh: ‘Fine’, with 2,539,148 (mostly tetrahedral) elements. Study: ‘Stationary’. Relative tolerance: 0.001. The algorithm implies a numerical solution for the coupled Reynold-averaged Navier-Stokes equation (RANS) and convective diffusion equations [12]. Note that the natural convection term due to buoyancy was ignored here since we focused on the situation when radon entry from the soil was primarily induced by wind. The equation for the transport of diluted species $c_{i}$ is as follows:

(2) $$\nabla \cdot \left(-D_{Rn-air}\nabla c_{i}\right)+\vec{u}\cdot \nabla c_{i}=R_{i}$$

The radioactive radon decay process was presented by a pseudo-reaction term $R_{i}$; here, $\vec{u}$ and $D_{Rn-air}$ denote air flow velocity and radon–air diffusivity.

Prior to simulation of the transport phenomena within the house depicted in the Figure 1, we tested the accuracy and validity of our algorithm with a case study [2], which reported in the results on radon dispersion in an empty closed model room with dimensions 3.01 m × 3.01 m × 3.0 m, in which the radon exhalation rate from the wall was determined by active and passive measurements.

**Figure 2:**
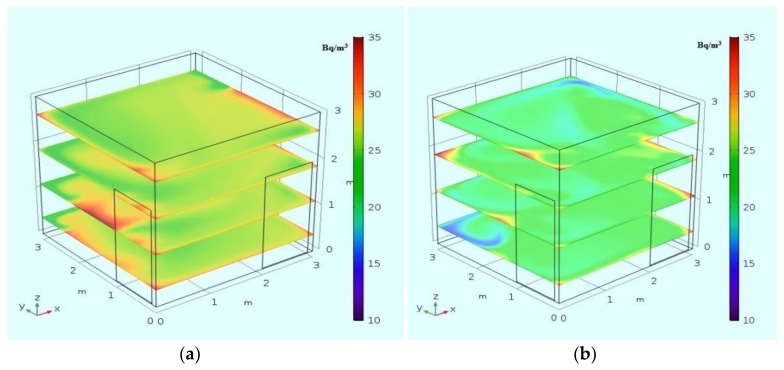
Radon concentration in the room for the case of exhalation of radon from the walls [2]. (**a**) laminar flow; (**b**) k-ε turbulence model.

The simulated results on radon concentration distribution at several levels in the above mentioned model room are shown in Figure 2. The selected representative values of radon concentrations are given in Table 1. Our CFD model outcomes are reasonably consistent with the results of Chauhan et al. [2]. Both studies indicate a certain non-uniformity of radon concentration fields and elevated values of radon concentration in the corners of the room.

Although the application of the laminar flow regime also yielded the realistic values of radon concentration, the turbulent flow mode has been employed in the following simulation to comply with the mixing mechanism of turbulence that occurs in regions with high values of velocity gradients.

## 5. Results and Discussion

**Figure 3:**
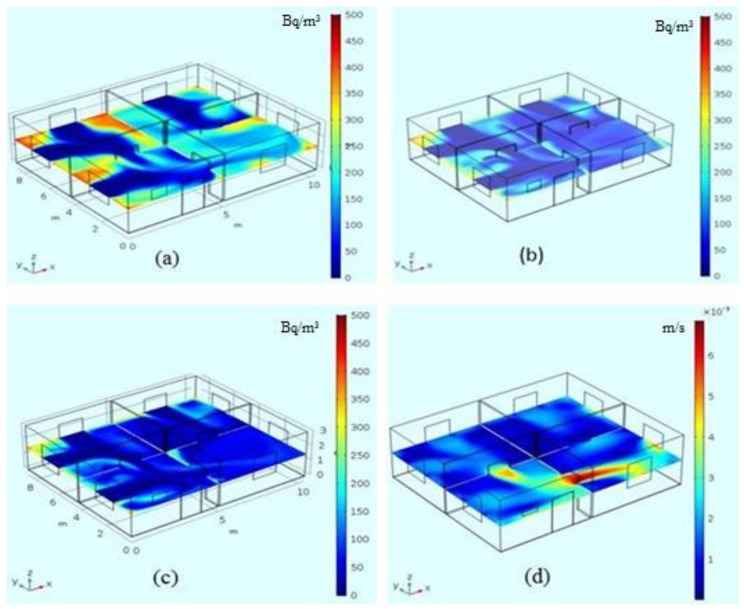
Radon concentration changes vs. air exchange rate, (**a**) λ = 0.17 h^−1^; (**b**) λ = 0.34 h^−1^; (**c**) λ = 0.51 h^−1^; and (**d**) velocity magnitude distribution for λ = 0.17 h^−1^, z = 1.5 m.

**Figure 4:**
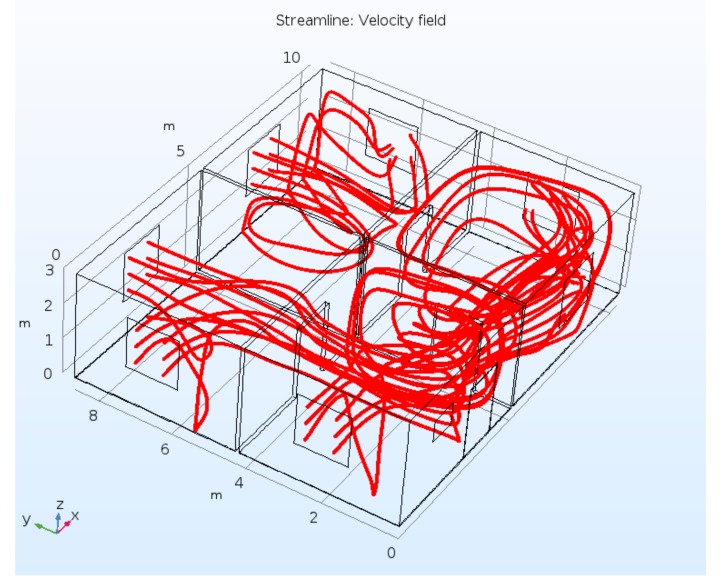
Velocity streamlines, λ = 0.17 h^−1^.

The calculated radon concentration fields at z = 1.5 m for λ = 0.17 h^−1^, 0.34 h^−1^, and 0.5 h^−1^ and the velocity magnitude field for the same plane for λ = 0.17 h^−1^ for the house model are presented in Figure 3. The selected data which demonstrate non-uniformity of the radon concentration are presented in Table 2. Although the model represents an empty house case, there are clear pronounced regions with elevated and reduced values of radon concentration. The elevated concentrations are mainly observed in the corners; the reduced values are located in the center of the rooms and, as expected, near the windows at the windward side. The structure of radon concentration field is obviously correlated with the pattern of the magnitude velocity; for example, the stagnation zones in the rooms at the windward side can be interpreted as being locked by neighboring airstreams emerging from windows. An additional illustration of the particulars of the convection diffusion mechanism is presented in Figure 4 which is a 3D structure of the streamlines in the house. High-density streamlined regions relate to above-average mixing or pumping work of the forced convection mechanism and correspond to the lower concentration of radon species.

From Table 2 it is interesting to observe that the reduction of the radon concentration with increasing air exchange rate (which in this case is equivalent to higher values of airtightness) does not occur evenly in the stagnation places (corners). This can be attributed to a different condition in order for separation to occur in these zones.

Some of the key parameters of the models are the inlet concentration of the radon in the radon-carrying airstream or the value of the radon exhalation flux. This paper’s results referred to the value of 500 Bq∙m^−3^; however, this might be much higher in some regions due to outcomes of the local underground radioactivity. Several studies [2,3,5,6] dealt with comparatively low or moderate levels of radon concentrations associated with emanation of radon from wall building materials when the radon transport is dominated by diffusion. This paper focused on modeling of the convective mechanism of penetration of radon into the building, i.e., on the transport of radon by pressure-driven flow, where the levels of radon concentration might be much higher [1,4,10].

Thus, we monitored in a parallel study the evolution of radon concentration vs. time in a basement room measuring 2.35 m × 2.5 m × 2.4 m. The room was located in the Kostanay region of Kazakhstan, which is characterized with a high level of release of radon from underground [10]. The radon concentrations were measured with the Russian-made Alpharad Plus (Ltd ‘NTM-Zashita’, Moscow city, Russia) system for monitoring of radon, thoron, and their airborne progenies.

**Figure 5:**
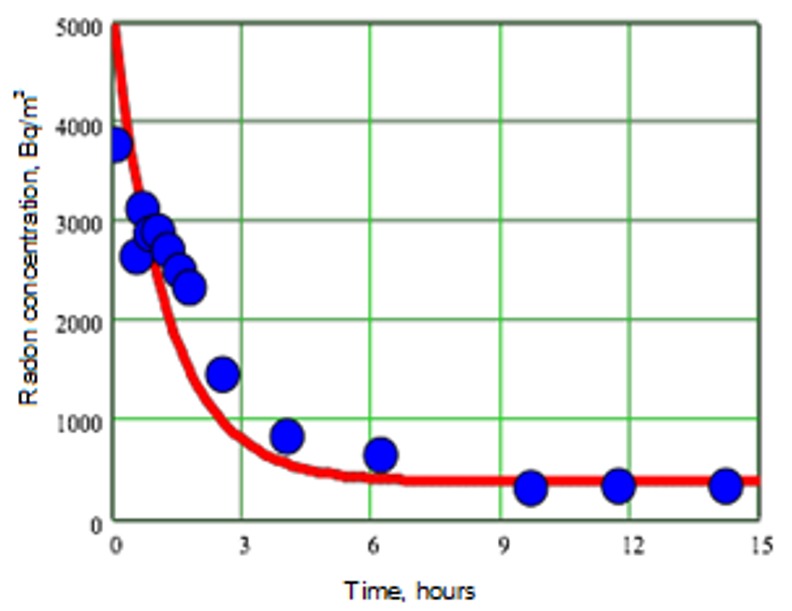
Change of the radon concentration in the basement with time upon activation of the ventilation of λ = 0.8 h^−1^.

Figure 5 shows the typical result of the measurement of the radon concentration vs. time under impact of the induced forced ventilation; the experiments were accomplished in the basement room in the Kostanay region of Kazakhstan. The application of a procedure based on Equation (1) [5] to this data yields the estimation of the radon entry rate *S* ≈ 300 Bq∙m^−3^ h^−1^. Inserting this value of radon volumetric source strengthens the radon species balance (Equation (3)):

(3) S×(volume of the room)=A×(walls and floor area)

which yields an apparent exhalation flux *A* from the walls of about 180 ± 25 Bq∙m^−2^ h^−1^. This is much higher than typical values of about 1.5 Bq∙m^−2^ h^−1^ for the wall concrete materials [2]. Therefore, we conclude that we witnessed the convective mechanism of radon entry.

## 6. Conclusions

The modeling of radon concentration distribution in this research demonstrates substantial non-uniformity of the radon concentration within the house, which should particularly be taken into consideration in regions with the potential for a high radon entry rate from the soil. Increase of the air exchange rate leads to levelling off of the radon concentration; however, such regulation is commonly is limited by energy resources.

## Figures and Tables

**Table 1:** Comparison of simulation results with study [2]; radon concentration at z = 1.22 m. CFD: computational fluid dynamics.

Location	Radon Concentration, Bq/m^3^				
	CFD [2]	Active Measurement [2]	Passive Measurement [2]	CFD Laminar Flow (This Paper)	CFD Turbulent Flow, k-ε Model (This Paper)
Corner 1	20	27	30	30.8	33.0
Corner 2	21	24	30	33.2	34.2
Corner 3	27	27	30	36.4	32.5
Corner 4	13	18	8	16.3	21
Center	23	22	42	21.5	26.8

**Table 2:** Radon concentration at the selected points in the windward corner room at z = 1.5 m.

Air Exchange Rate, h^−1^	Radon Concentration, Bq/m^3^					
	Corner 1	Corner 2	Corner 3	Corner 4	Center	House Volume Average
0.17	338.9	444.9	417.2	269.8	13.5	181.25
0.34	304.9	416.8	276.9	22.0	12.4	106.9
0.51	296.0	389.2	256.8	12.1	12.6	77.9
